# Treatment Patterns and Healthcare Resource Utilization among Patients with Advanced or Metastatic Soft Tissue Sarcoma in US Community Practices

**DOI:** 10.1155/2020/1765319

**Published:** 2020-02-28

**Authors:** Eric Nadler, Kathleen Aguilar, Chuck Wentworth, Marley Boyd, E. Susan Amirian, Scott Barker, Pearl French, Thomas Wilson, Lisa M. Hess

**Affiliations:** ^1^Texas Oncology, Dallas, TX 75246, USA; ^2^McKesson Life Sciences, The Woodlands, TX 77380, USA; ^3^Eli Lilly and Company, Indianapolis, IN 46285, USA

## Abstract

**Introduction:**

This study was designed to describe demographic and clinical characteristics of patients diagnosed with advanced or metastatic soft tissue sarcoma (STS) and to examine treatment and healthcare resource utilization patterns of this patient population in a United States (US) community-based oncology practice setting over time.

**Methods and Materials:**

A retrospective observational study was conducted within the US Oncology Network (USON). Patients were eligible if they were diagnosed with advanced or metastatic STS and were treated at a USON site between 01 July 2015 and 31 August 2018. Demographic, clinical, and treatment characteristics were described for the overall study population. Comparisons between patients by time period (prior to and after October 2016) were evaluated using the *T* test for continuous variables and chi-squared test for categorical variables. Data were available for analysis through 31 August 2018.

**Results:**

Demographic and clinical characteristics of the eligible study cohort (*N* = 376) were similar between patients who initiated treatment before and after October 2016 (all *p* > 0.05). Forty-three unique regimens were observed in the first-line setting, with the predominant regimen (gemcitabine + docetaxel) received by 33.2% (*n* = 125) patients. Prior to October 2016, 45.4% of patients received first-line gemcitabine + docetaxel, while 29.0% received this regimen after October 2016.

**Conclusions:**

While demographic and clinical characteristics were similar, treatment patterns changed in 2016. Future research should evaluate the impact of changing drug approvals and clinical trial results on future treatment patterns.

## 1. Introduction

In the United States (US), soft tissue sarcoma (STS) is considered a relatively rare cancer, accounting for fewer than 1% of all malignancies [1, 2]. Fewer than 15,000 new cases and approximately 5,000 related deaths were expected in 2018 [2]. STSs arise from mesenchymal tissue and can develop at any site in the body but most frequently present in the extremities, trunk and retroperitoneum, head, or neck [3–5]. More than 50 distinct subtypes of STS have been identified, with the most common being undifferentiated pleomorphic sarcoma, gastrointestinal stromal tumors (GIST), liposarcoma, and leiomyosarcoma [6, 7].

For patients with advanced, unresectable or metastatic non-GIST STS, systemic chemotherapy, particularly containing anthracyclines, has been the standard of care for several decades [1, 5, 8]. The treatment landscape for advanced and metastatic STS is changing, however, with the recent approvals of novel targeted and biologic therapies. As of January 2020, 6 drugs have US Food and Drug Administration (FDA) approval for the treatment of non-GIST STS: dactinomycin, doxorubicin hydrochloride, eribulin mesylate, imatinib mesylate, pazopanib hydrochloride, and trabectedin [9].

The National Comprehensive Cancer Network (NCCN) guidelines (4.2019) recommend over 20 different regimens for the treatment of STS with nonspecific histologies [1]. Most of these regimens were given a category 2A recommendation, meaning that the NCCN committee recommends the treatments based on lower-level evidence. Trabectedin, however, was given a category 1 recommendation for treatment of liposarcoma and leiomyosarcoma (L-Types) and eribulin for liposarcoma.

A high degree of treatment heterogeneity has been observed in the previous observational research of patients diagnosed with STS in the US. Based on a medical record review of 99 patients with metastatic or relapsed STS treated in a tertiary academic cancer care center between 2001 and 2011, most patients received anthracycline- or gemcitabine-based regimens, but there was not a predominant treatment in any line of therapy [10]. Similarly, Villalobos et al. (2017) performed a retrospective study of 2006–2015 claims data from a large US health insurance plan and reported that no single treatment regimen was received by more than 31% of the patient population in the first-through fourth-line settings [11].

Olaratumab is a monoclonal antibody that selectively binds platelet-derived growth factor receptor alpha and blocks ligand binding [12]. On 19 October 2016, the FDA granted accelerated approval to olaratumab for use with doxorubicin for the treatment of patients with STS who cannot be cured with radiation or surgery and have a type of STS for which an anthracycline-containing regimen is appropriate. The accelerated approval was based on phase II trial results that demonstrated a median overall survival of 26.5 months among patients with metastatic STS treated with olaratumab and doxorubicin combination therapy, compared with 14.7 months among patients receiving doxorubicin alone [13].

The clinical benefit of olaratumab and doxorubicin, however, was not confirmed in the subsequent phase III ANNOUCE trial [14]. In this trial, patients with unresectable locally advanced or metastatic STS were randomized to receive doxorubicin with olaratumab or placebo. No significant differences were observed in median overall survival between the groups, both in the full study population or in the subpopulation of patients with leiomyosarcoma. Median progression-free survival was lower among patients who received olaratumab compared with those who received placebo (*P* = 0.04). As such, in January 2019, the company announced that the phase III trial did not confirm the clinical benefit of olaratumab in combination with doxorubicin as compared with doxorubicin alone, and it was subsequently withdrawn from the market.

This study was designed to describe current clinical and demographic characteristics, treatment patterns, and healthcare resource utilization (HCRU) among patients diagnosed with advanced or metastatic STS in a US community-based network of oncology practices. These factors were compared among the cohorts treated prior to and following the October 2016 FDA-accelerated approval of olaratumab.

## 2. Methods

### 2.1. Setting and Data Source

A retrospective observational cohort study was conducted of adult patients newly diagnosed with advanced or metastatic STS within the US Oncology Network (USON). The USON is a network of more than 450 community-based oncology clinics across the US [15]. More than 1,400 physicians are affiliated with the USON, which treats nearly 1,000,000 patients annually. The study protocol was granted an exception and waiver of informed consent by the US Oncology Institutional Review Board.

Both structured and unstructured variables from the USON's electronic health record (EHR) and targeted chart review were used to build the study database. The EHR of the USON, iKnowMed (iKM), captures outpatient practice encounter histories for patients under community-based care, including but not limited to: patient demographics such as age and gender; clinical information such as disease diagnosis, diagnosis stages, performance status information, and laboratory testing results; and treatment information, such as line of therapy and treatment administrations within the USON. The claims and remittance database of the USON was used to assess HCRU of services provided within the USON.

A targeted chart review was performed to capture information on key variables that were anticipated to be poorly documented in the structured EHR fields. This included capture of some data only available in unstructured fields, including information on hospitalizations and emergency department (ED) visits that occurred during the study observation period, as well as histological subtypes and tumor location. Chart review was accomplished by use of a secure, web-based electronic case report form and conducted by trained oncology professionals. These chart reviewers captured information as it was explicitly documented in the medical record; central pathology review was not undertaken to verify what physicians recorded in the patient record. The chart review was conducted for a subset of eligible patients who initiated first-line treatment between 01 July 2015 and 31 August 2017.

### 2.2. Study Population

Eligible patients were at least 18 years of age with a documented diagnosis of STS and who initiated first-line treatment between 01 July 2015 and 31 August 2018. Additionally, patients were required to have at least two follow-up visits within the USON after first-line treatment initiation at clinics that had fully implemented the EHR system, iKM. Patients enrolled in clinical trials or who were diagnosed with another primary cancer were excluded from the analysis. Additional exclusion criteria included the following: diagnosis of Ewing's sarcoma, osteosarcoma or Kaposi's sarcoma, and receipt of treatments suggestive of clinical trial participation or nonsarcoma diagnosis (i.e., thalidomide, rituximab, afatinib, binimetinib, dabrafenib, regorafenib, and ribociclib).

### 2.3. Study Observation Period

Patients were followed from initiation of first-line treatment until the end of the study period (31 August 2018) or last USON visit date. All study variables and outcomes were assessed regardless of maximum follow-up using data available until the end of the study period. Baseline variables were assessed for the 60-day period prior to and up to 30 days after first-line treatment initiation. Patients were categorized in the pre-October 2016 cohort if they initiated first-line treatment between 01 July 2015 and 18 October 2016, and in the post-October 2016 cohort if they initiated treatment between 19 October 2016 and 31 August 2018. If a patient was included in the pre-October 2016 cohort but advanced to second-line treatment after 19 October 2016, they remained classified in the pre-October 2016 cohort.

### 2.4. Statistical Analysis

Descriptive statistics were used to examine demographic, clinical, and treatment characteristics for patients in the first- and second-line settings. Categorical variables (e.g., gender and performance status) were reported as frequencies and percentages. Continuous variables such as age were reported as mean, standard deviation, median, and range. In the case of missing observations, the number and percentage of missing values were reported. Programmatic logic was applied to categorize therapy sequences across lines of therapy based on start and stop dates, as well as the predefined line of therapy indicator in iKM.

Chi-squared or Fisher's exact test was used to assess differences between categories of variables when at least 5 patients were represented in each group. *F* test, *T* test, or Kruskal–Wallis test (for non-normally distributed data) were used for continuous variables. An alpha level of less than 0.05 was the primary criterion for statistical significance in this study. Results were compared between the pre- and post-October 2016 cohorts.

## 3. Results

### 3.1. Patient Characteristics

In total, 376 patients met eligibility criteria and were included in the analysis; 211 of these patients were selected for chart review, while the remaining 165 patients contributed to structured data only. Among the overall study population, 97 patients initiated first-line treatment in the pre-October 2016 period and 279 in the post-October 2016 period (prior to and after accelerated approval of olaratumab; Table 1). Of these patients, 196 received second-line therapy, with 77 receiving second-line in the pre-October 2016 period and 119 in the post-October 2016 period.

The median duration of follow-up across the study population was 7.3 months (range 0.0, 79.6; data not shown). Patients who initiated first-line treatment during the pre-October 2016 period had a median duration of follow-up of 12.5 months (range 0.0, 79.6) compared with a median duration of 5.6 months (range 0.0, 22.8) among patients who initiated first-line treatment during the post-October 2016 period.

Baseline demographic and clinical characteristics of patients who initiated first-line treatment and those who received second-line treatment are presented in Table 1. At initiation of first-line treatment, the median age of the study population was 62 years (range 20, 90+) with 48.4% male and 76.3% Caucasian. Approximately 75% of the population had an Eastern Cooperative Oncology Group (ECOG) performance status score of 0–1. The majority of patients were reported as having sarcoma without further diagnostic data (56.6%), followed by leiomyosarcoma (22.1%) and uterine sarcoma (13.8%). More than 60% of patients had evidence of metastases at diagnosis and 39.6% of patients had lung metastases at baseline.

Tumor histology and location were not documented in structured fields of the EHR and, as such, were only captured for patients selected for chart review (Table 1). Among these 211 patients, the most common histological subtype was leiomyosarcoma (*n* = 87), followed by undifferentiated pleomorphic sarcoma (*n* = 36), liposarcoma (*n* = 16), and angiosarcoma (*n* = 11), with the remaining each having 10 or fewer patients. Forty-seven patients had lower limb tumors, 45 uterine tumors, 20 retroperitoneal tumors, and 16 upper limb tumors, with 10 or fewer patients having tumors in each of the other documented locations.

Among the 196 patients who received second-line treatment, at initiation of first-line treatment the median age was 60 years (range 21, 86), with 45.9% male and 73.5% Caucasian (Table 1). Approximately 78% had an ECOG performance status score of 0–1. Patients who initiated second-line treatment were reported as having sarcoma without further diagnostic data (54.6%), leiomyosarcoma (23.0%), or uterine sarcoma (15.3%). More than 70% of patients who received second-line therapy had documented metastases at baseline.

Overall, demographic and clinical characteristics were similar between patients in the pre- and post-October 2016 cohorts (Table 1). Evidence of metastasis was the only statistical difference observed: a higher proportion of patients who initiated first-line treatment in the pre-October 2016 period had documented metastases compared with those who initiated treatment in the post-October 2016 period (83.5% vs. 56.6%, respectively; *P* < 0.0001). A higher proportion of the patients in the pre-October 2016 cohort had documented bone, lymph node, and lung metastases than in the post-October 2016 cohort (*P* < 0.05 for all). In the second-line setting, a higher proportion of the patients in the pre-October 2016 cohort had lung metastases (*P*=0.0183); no other significant differences were observed between the groups.

### 3.2. Treatment Patterns

Table 2 presents the treatment regimen distributions observed in this study. Forty-three unique regimens were observed in the first-line setting, 38 in the second-line setting and 31 in the third-line setting both before and after October 2016. Across the study period, both before and after October 2016, gemcitabine + docetaxel was the most common first-line regimen (*n* = 125, 33.2%), followed by olaratumab + doxorubicin (*n* = 64, 17.0%), gemcitabine (*n* = 27, 7.2%), doxorubicin + ifosfamide (*n* = 25, 6.6%), and doxorubicin monotherapy (*n* = 24. 6.4%), with each of the remaining first-line regimens received by fewer than 5% of the study population. Fifty patients (13.3%) received a first-line regimen classified into an “other” category because it was received by fewer than 5 patients total (in the first-line setting, there were 33 unique regimens grouped into this category).

During the pre-October 2016 period, 45.4% (*n* = 44) of patients received first-line gemcitabine + docetaxel, 13.4% (*n* = 13) doxorubicin, and 7.2% (*n* = 7) liposomal doxorubicin (Table 2). Among those who initiated first-line treatment in the post-October 2016 period, 29.0% (*n* = 81) received gemcitabine + docetaxel, 22.9% (*n* = 64) olaratumumab + doxorubicin, and 7.9% (*n* = 22) gemcitabine.

In total, 180 patients (47.9%) did not receive second-line treatment and 291 (77.4%) did not receive third-line treatment during the study period within the USON (Table 2). Including the patients who did not proceed to second-line treatment, 117 unique first-to second-line treatment sequences were observed across both time periods. Among patients who initiated first-line treatment prior to October 2016, 55 unique sequences were observed, while 85 unique sequences were observed among patients who initiated first-line treatment after October 2016. Among patients who received a second-line treatment, the most common first-to second-line treatment sequence observed was gemcitabine + docetaxel, followed by olaratumab + doxorubicin (observed for 5.2% (*n* = 5) patients in the pre-October 2016 cohort and 5.0% (*n* = 14) in the post-October 2016 cohort).

**Figure 1:**
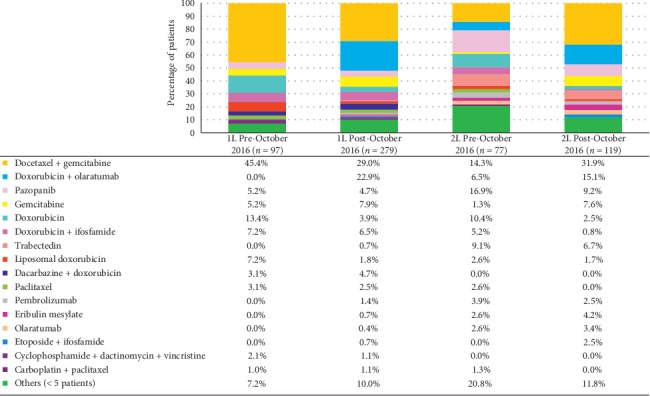
First- and second-line treatment regimens in the pre- and post-October 2016 periods received by at least 5 patients.

During the post-October 2016 period, 111 patients in the study dataset received olaratumab either alone or in combination with other agents in the first- through third-line treatment settings. A small proportion of patients (6.5%) who initiated first-line treatment in the pre-October 2016 period proceeded to receive second-line olaratumab + doxorubicin during the post-October 2016 period (Figure 1).

Among patients who initiated first-line treatment in the post-October 2016 period, 22.9% and 15.1% received olaratumab + doxorubicin in the first- and second-line setting, respectively (Figure 1). The proportion of patients who received gemcitabine + docetaxel was higher among patients who initiated first-line treatment in the pre- versus post-October 2016 period (45.4% vs. 29.0%, respectively). In contrast, the proportion of patients who received gemcitabine + docetaxel was higher among those who initiated second-line treatment in the post-October 2016 period (31.9% vs. 14.3%, respectively).

### 3.3. Healthcare Resource Utilization (HCRU)

Among the 211 patients who underwent a chart review, hospitalizations were documented for 116 (55.0%), ED visits for 83 (39.3%) and surgeries for 42 (19.9%; Table 3). In both the first- and second-line settings, a significantly higher proportion of patients in the pre-October 2016 cohort had documentation of these HCRU events compared with the post-October 2016 cohort (*P* < 0.01 for all).

Patients in the pre-October 2016 cohort had similar utilization of outpatient visits and laboratory procedures compared with those in the post-October 2016 cohort (Table 3). The use of granulocyte-colony stimulating factor (G-CSF) use was significantly higher in the pre-October 2016 cohort (*P* < 0.0001 for both first- and second-line). Across the entire study cohort, 3.5% had a record of dexrazoxane use.

## 4. Discussion

The results of this study provide insight into the patient characteristics and treatment patterns of patients diagnosed with advanced or metastatic STS receiving care in a US community-based practice setting before and shortly after the time of the accelerated approval of olaratumab on 19 October 2016. At initiation of first-line treatment, the baseline characteristics of the study population were similar to those observed in other real-world studies [10, 11, 16]. Specifically, the median age of the study population (60 years) and proportion of male patients were consistent with other studies that have reported median ages ranging from 52 to 62 years, with approximately 50% being male. Leiomyosarcoma and undifferentiated pleomorphic sarcoma were the most common histological subtypes observed in this study, which is consistent with other published accounts of histological frequency [6, 7]. A higher proportion of patients who initiated treatment in the pre-October 2016 period had evidence of metastases; otherwise, no meaningful differences were observed in the baseline demographic and clinical characteristics among patients who initiated first-line treatment before and after October 2016.

As observed in previously published observational studies of patients with advanced or metastatic STS, a high degree of treatment heterogeneity was observed across lines of therapy in this study [10, 11, 17, 18]. In this study, 43 unique regimens were observed with 117 first-to second-line treatment sequences. Similar to what was reported by Villalobos et al. (2017), fewer than 35% of patients received the most common regimen (gemcitabine + docetaxel) [11]. The observed diversity in treatment approaches observed in STS is likely due to both underlying differences in disease characteristics and lack of predominant guideline regimen recommendations [1, 19, 20]. Future studies should consider the impact of this treatment heterogeneity on clinical outcomes and how treatment algorithms may aid in provider decision-making.

During the approximate 2-year period following the accelerated approval of olaratumab, 111 patients (29.5%) received olaratumab in the first-, second- or third-line settings. Among patients who initiated first-line treatment during the pre-October 2016 period, 45% received first-line gemcitabine + docetaxel. During the post-October 2016 period, first-line use of gemcitabine + docetaxel was 29% and 23% received olaratumab + doxorubicin. These findings suggest that olaratumab was being utilized in community-based practice settings for the care of patients with advanced or metastatic STS. Given the limited follow-up period subsequent to olaratumab approval, the data were not mature enough to support survival analyses. Sufficient data were not available to examine the shift in treatment patterns following the results of the phase III study.

The GeDDiS trial was published during the observation period of this study and may have influenced the lower use of first-line gemcitabine + docetaxel in the post-October 2016 period [21]. In the GeDDis trial, patients with locally advanced or metastatic STS were randomized to receive first-line doxorubicin or gemcitabine + docetaxel. While no survival or progression-free survival differences were found between the groups, patients who received gemcitabine + docetaxel experienced more dose modifications and treatment discontinuation due to toxicity, as well as lower quality of life scores. While the GeDDiS trial did not report a statistically significant difference in progression-free survival between the treatment arms, some physicians may have interpreted this as meaning gemcitabine + docetaxel was still a viable treatment option [22].

Approximately 50% of patients did not receive second-line treatment in the USON database; however, this could in part be due to limited follow-up on the study population. The analyses did not account for patients who had recently discontinued first-line therapy, were still receiving first-line therapy at the end of the database, or who sought additional treatment outside of the USON. With similar limitations, approximately 60% of those treated in the second-line setting did not receive third-line therapy in the database.

Compared with patients who initiated first-line treatment prior to the October 2016 approval of olaratumab, those who initiated treatment in the post-October 2016 period had fewer observed hospitalizations, ED visits, and surgeries documented in the EHR (*P* < 0.01 for all), which could be due to approximately 7 months of additional follow-up among patients who initiated treatment during the pre-October 2016 period (median 12.5 vs. 5.6 months of follow-up).

Data for this study were sourced from the USON's EHR database, which contains information documented during the course of routine patient care and was not collected for research purposes. Consequently, data entry errors and incomplete records could neither be verified nor corrected. Certain variables of interest were inconsistently documented. To enhance the study dataset, a chart review was performed to capture information that was not well-represented in the structured fields of the EHR, including histology and tumor location, for a subset of the study population. Likewise, information for services performed outside of the USON, such as hospitalizations and ED visits, was restricted to records and progress notes and could only be captured for patients undergoing chart review. By sourcing data solely from USON that utilized the full EHR capacities of iKM, the generalizability of this study may also be limited, and the cohort cannot be generalized to the overall US population.

## 5. Conclusion

This real-world retrospective study of patients with advanced or metastatic STS treated in a large community-based oncology network provides data regarding patient characteristics and treatment patterns outside of a clinical trial setting. The demographic and clinical data observed were consistent with those of the previous studies. The treatment pattern data suggest a dynamic treatment landscape that changed following the accelerated approval of olaratumab. Despite the increased utilization of olaratumab plus doxorubicin during the study period, considerable heterogeneity in treatment regimens was observed across the lines of therapy, suggesting the need for more evidence-based decision support to facilitate the care of the patient with STS.

## Figures and Tables

**Table 1:** Baseline demographic and clinical characteristics overall and for the pre- and post-2016 cohorts.

	Overall (*n* = 376)	1L pre-October 2016 cohort (*n* = 97)	1L post-October 2016 cohort (*n* = 279)	*P* value	Overall (*n* = 196)	2L pre-October 2016 cohort (*n* = 77)	2L post-October 2016 cohort (*n* = 119)	*P* value
Median age at 1L initiation *(years, min, max)*	62 (20, 90+)	62 (25, 84)	62 (20, 90+)	0.9460	60 (21, 86)	60 (30, 82)	61 (21, 86)	0.8195
*Male*, *n (%)*	182 (48.4)	50 (51.5)	132 (47.3)	0.4722	90 (45.9)	38 (49.4)	52 (43.7)	0.4380
*Race*, *n (%)*				0.8902				0.8028
Caucasian	28 (76.3)	74 (76.3)	213 (76.3)		144 (73.5)	56 (72.7)	88 (74.0)	
Black or African American	27 (7.2)	6 (6.2)	21 (7.5)		16 (8.2)	6 (7.8)	10 (8.4)	
Others	15 (4.0)	5 (5.2)	10 (3.6)		9 (4.6)	5 (6.5)	4 (3.4)	
No information	47 (12.5)	12 (12.4)	35 (12.5)	—	27 (13.8)	10 (13.0)	17 (14.3)	—
*BMI*, *n (%)*				0.7285				0.8281
Underweight (BMI < 18.5)	9 (2.4)	1 (1.0)	8 (2.9)		5 (2.6)	1 (1.3)	4 (3.4)	
Normal (BMI: 18.5 - < 25)	104 (27.7)	27 (27.8)	77 (27.6)		50 (25.5)	20 (26.0)	30 (25.2)	
Overweight (BMI: 25– < 30)	116 (30.9)	33 (34.0)	83 (29.7)		56 (28.6)	24 (31.2)	32 (26.9)	
Obese (BMI = 30+)	147 (39.1)	36 (37.1)	111 (39.8)		85 (43.4)	32 (41.6)	53 (44.5)	
*ECOG score*, *n (%)*				0.8993				0.8161
0	62 (16.5)	17 (17.5)	45 (16.1)		38 (19.4)	15 (19.5)	23 (19.3)	
1	221 (58.8)	57 (58.8)	164 (58.8)		116 (59.2)	45 (58.4)	71 (59.7)	
2	39 (10.4)	8 (8.2)	31 (11.1)		13 (6.6)	7 (9.1)	6 (5.0)	
3	2 (0.5)	0 (0.00)	2 (0.7)		1 (0.5)	0 (0.00)	1 (0.8)	
No information	52 (13.8)	15 (15.5)	37 (13.3)	—	28 (14.3)	10 (13.0)	18 (15.1)	—
*Diagnosis*, *n (%)*				0.6024				0.3299
Sarcoma	213 (56.6)	56 (57.7)	157 (56.3)		107 (54.6)	41 (53.2)	66 (55.5)	
Leiomyosarcoma	83 (22.1)	25 (25.8)	58 (20.8)		45 (23.0)	23 (29.9)	22 (18.5)	
Uterine sarcoma	52 (13.8)	12 (12.4)	40 (14.3)		30 (15.3)	10 (13.0)	20 (16.8)	
Fibrosarcoma	14 (3.7)	1 (1.0)	13 (4.7)		8 (4.1)	1 (1.3)	7 (5.9)	
Chondrosarcoma	8 (2.1)	2 (2.1)	6 (2.2)		3 (1.5)	1 (1.3)	2 (1.7)	
Rhabdomyosarcoma	6 (1.6)	1 (1.0)	5 (1.8)		3 (1.5)	1 (1.3)	2 (1.7)	
*Histology* ^*∗*^, *n (%)*				0.1641				0.1871
Angiosarcoma	11 (2.9)	8 (8.2)	3 (1.1)		7 (3.6)	6 (7.8)	1 (0.8)	
Fibroblastic/myofibroblastic	10 (2.7)	2 (2.1)	8 (2.9)		7 (3.6)	2 (2.6)	5 (4.2)	
Leiomyosarcoma	87 (23.1)	40 (41.2)	47 (16.8)		62 (31.6)	35 (45.5)	27 (22.7)	
Liposarcoma	16 (4.3)	4 (4.1)	12 (4.3)		9 (4.6)	3 (3.9)	6 (5.0)	
Nerve sheath sarcoma	4 (1.1)	2 (2.1)	2 (0.7)		1 (0.5)	0 (0.00)	1 (0.8)	
Others	25 (6.6)	7 (7.2)	18 (6.5)		15 (7.7)	4 (5.2)	11 (9.2)	
Rhabdomyosarcoma	10 (2.7)	5 (5.2)	5 (1.8)		7 (3.6)	4 (5.2)	3 (2.5)	
Synovial	8 (2.1)	4 (4.1)	4 (1.4)		6 (3.1)	3 (3.9)	3 (2.5)	
Undifferentiated pleomorphic sarcoma	36 (9.6)	19 (19.6)	17 (6.1)		24 (12.2)	14 (18.2)	10 (8.4)	
Documented unknown	4 (1.1)	2 (2.1)	2 (0.7)	—	3 (1.5)	2 (2.6)	1 (0.8)	—
No information	165 (43.9)	4 (4.1)	161 (57.7)	—	55 (28.1)	4 (5.2)	51 (42.9)	—
*Tumor location* ^*∗*^, *n (%)*				0.5625				0.8542
Axilla	1 (0.3)	1 (1.0)	0 (0.00)		1 (0.5)	1 (1.3)	0 (0.00)	
Breast	2 (0.5)	2 (2.1)	0 (0.00)		2 (1.0)	2 (2.6)	0 (0.00)	
Genitourinary	5 (1.3)	2 (2.1)	3 (1.1)		5 (2.6)	2 (2.6)	3 (2.5)	
Head or neck	8 (2.1)	5 (5.2)	3 (1.1)		4 (2.0)	3 (3.9)	1 (0.8)	
Lower limb	47 (12.5)	24 (24.7)	23 (8.2)		33 (16.8)	18 (23.4)	15 (12.6)	
Mediastinum, lung, pleura	5 (1.3)	3 (3.1)	2 (0.7)		4 (2.0)	3 (3.9)	1 (0.8)	
Others	44 (11.7)	19 (19.6)	25 (9.0)		29 (14.8)	13 (16.9)	16 (13.4)	
Pelvis	5 (1.3)	2 (2.1)	3 (1.1)		4 (2.0)	2 (2.6)	2 (1.7)	
Retroperitoneal	20 (5.3)	8 (8.2)	12 (4.3)		11 (5.6)	7 (9.1)	4 (3.4)	
Trunk	10 (2.7)	4 (4.1)	6 (2.2)		6 (3.1)	3 (3.9)	3 (2.5)	
Upper limb	16 (4.3)	5 (5.2)	11 (3.9)		10 (5.1)	4 (5.2)	6 (5.0)	
Uterus	45 (12.0)	16 (16.5)	29 (10.4)		29 (14.8)	13 (16.9)	16 (13.4)	
Documented unknown	3 (0.8)	2 (2.1)	1 (0.4)	—	3 (1.5)	2 (2.6)	1 (0.8)	—
No information	165 (43.9)	4 (4.1)	161 (57.7)	—	55 (28.1)	4 (5.2)	51 (42.9)	—
*Tumor grade*, *n (%)*				0.5287				0.4477
Well differentiated	17 (4.5)	4 (4.1)	13 (4.7)		6 (3.1)	2 (2.6)	4 (3.4)	
Moderately differentiated	47 (12.5)	15 (15.5)	32 (11.5)		27 (13.8)	13 (16.9)	14 (11.8)	
Poorly differentiated	158 (42.0)	45 (46.4)	113 (40.5)		83 (42.3)	36 (46.8)	47 (39.5)	
Undifferentiated	5 (1.3)	1 (1.0)	4 (1.4)		2 (1.0)	0 (0.00)	2 (1.7)	
No information	149 (39.6)	32 (33.0)	117 (41.9)	—	78 (39.8)	26 (33.8)	52 (43.7)	—
*Count of metastatic sites*, *n (%)*				<0.0001				0.3188
0	137 (36.4)	16 (16.5)	121 (43.4)		54 (27.6)	16 (20.8)	38 (31.9)	
1	127 (33.8)	40 (41.2)	87 (31.2)		73 (37.2)	28 (36.4)	45 (37.8)	
2	71 (18.9)	24 (24.7)	47 (16.8)		46 (23.5)	20 (26.0)	26 (21.8)	
3	29 (7.7)	11 (11.3)	18 (6.5)		15 (7.7)	8 (10.4)	7 (5.9)	
4	7 (1.9)	3 (3.1)	4 (1.4)		3 (1.5)	2 (2.6)	1 (0.8)	
5	5 (1.3)	3 (3.1)	2 (0.7)		5 (2.6)	3 (3.9)	2 (1.7)	
*Bone*, *n (%)*	46 (12.2)	18 (18.6)	28 (10.0)	0.0274	28 (14.3)	14 (18.2)	14 (11.8)	0.2099
*Bowel*, *n (%)*	4 (1.1)	1 (1.0)	3 (1.1)	1.0000	2 (1.0)	1 (1.3)	1 (0.8)	1.0000
*Brain*, *n (%)*	3 (0.8)	0 (0.00)	3 (1.1)	0.5720	1 (0.5)	0 (0.00)	1 (0.8)	1.0000
*Colon*, *n (%)*	3 (0.8)	1 (1.0)	2 (0.7)	1.0000	1 (0.5)	1 (1.3)	0 (0.00)	0.3929
*Lymph node*, *n (%)*	37 (9.8)	17 (17.5)	20 (7.2)	0.0032	20 (10.2)	10 (13.0)	10 (8.4)	0.3005
*Soft tissue*, *n (%)*	18 (4.8)	7 (7.2)	11 (3.9)	0.1933	12 (6.1)	5 (6.5)	7 (5.9)	0.8616
*Liver*, *n (%)*	47 (12.5)	16 (16.5)	31 (11.1)	0.1673	30 (15.3)	14 (18.2)	16 (13.4)	0.3684
*Lung*, *n (%)*	149 (39.6)	54 (55.7)	95 (34.1)	0.0002	89 (45.4)	43 (55.8)	46 (38.7)	0.0183
*Skin*, *n (%)*	8 (2.1)	3 (3.1)	5 (1.8)	0.4303	4 (2.0)	3 (3.9)	1 (0.8)	0.3019
*Other sites of*, *n (%)*	94 (25.0)	31 (32.0)	63 (22.6)	0.0661	60 (30.6)	24 (31.2)	36 (30.3)	0.8918

1L, first-line; 2L, second-line; BMI, body mass index; ECOG, Eastern Cooperative Oncology Group; Min, minimum; Max, maximum; SD, standard deviation. ^*∗*^Variable assessed among patients selected for chart review (*n* = 211).

**Table 2:** Treatment regimens in the pre- and post-2016 periods.

	Overall (*n* = 376)	1L pre-October 2016 cohort (*n* = 97)	1L post-October 2016 cohort (*n* = 279)
*1L regimen distribution*, *n (%)*			
Gemcitabine + docetaxel	125 (33.2)	44 (45.4)	81 (29.0)
Olaratumab + doxorubicin	64 (17.0)	0 (0.0)	64 (22.9)
Gemcitabine	27 (7.2)	5 (5.2)	22 (7.9)
Doxorubicin + ifosfamide	25 (6.6)	7 (7.2)	18 (6.5)
Doxorubicin	24 (6.4)	13 (13.4)	11 (3.9)
Pazopanib	18 (4.8)	5 (5.2)	13 (4.7)
Dacarbazine + doxorubicin	16 (4.3)	3 (3.1)	13 (4.7)
Liposomal doxorubicin	12 (3.2)	7 (7.2)	5 (1.8)
Paclitaxel	10 (2.7)	3 (3.1)	7 (2.5)
Cyclophosphamide + dactinomycin + vincristine	5 (1.3)	2 (2.1)	3 (1.1)
Other 1L regimen^*∗*^	50 (13.3)	8 (8.2)	42 (14.1)
*2L regimen distribution*, *n (%)*			
No 2L treatment observed	180 (47.9)	20 (20.6)	160 (57.3)
Gemcitabine + docetaxel	49 (13.0)	11 (11.3)	38 (13.6)
Pazopanib	24 (6.4)	13 (13.4)	11 (3.9)
Olaratumab + doxorubicin	23 (6.1)	5 (5.2)	18 (6.5)
Trabectedin	15 (4.0)	7 (7.2)	8 (2.9)
Doxorubicin	11 (2.9)	8 (8.2)	3 (1.1)
Gemcitabine	10 (2.7)	1 (1.0)	9 (3.2)
Eribulin mesylate	7 (1.9)	2 (2.1)	5 (1.8)
Olaratumab	6 (1.6)	2 (2.1)	4 (1.4)
Pembrolizumab	6 (1.6)	3 (3.1)	3 (1.1)
Doxorubicin + ifosfamide	5 (1.3)	4 (4.1)	1 (0.4)
Other 2L regimen^*∗*^	40 (10.6)	21 (21.6)	19 (6.8)
*3L regimen distribution*, *n (%)*			
No 3L treatment observed	291 (77.4)	56 (57.7)	235 (84.2)
Olaratumab + doxorubicin	17 (4.5)	7 (7.2)	10 (3.6)
Trabectedin	15 (4.0)	9 (9.3)	6 (2.2)
Pazopanib	7 (1.9)	3 (3.1)	4 (1.4)
Gemcitabine + docetaxel	6 (1.6)	2 (2.1)	4 (1.4)
Other 3L regimen^*∗*^	40 (10.6)	20 (20.6)	20 (7.2)
*1L to 2L treatment sequence distribution*, *n (%)*			
Gemcitabine + docetaxel > none	60 (16.0)	9 (9.3)	51 (18.3)
Gemcitabine + docetaxel > olaratumab + doxorubicin	19 (5.1)	5 (5.2)	14 (5.0)
Gemcitabine + docetaxel > pazopanib	11 (2.9)	7 (7.2)	4 (1.4)
Gemcitabine + docetaxel > doxorubicin	8 (2.1)	6 (6.2)	2 (0.7)
Gemcitabine + docetaxel > trabectedin	8 (2.1)	4 (4.1)	4 (1.4)
Other 1L to 2L treatment sequence^†^	270 (71.8)	66 (68.0)	204 (73.1)

1L, first-line; 2L, second-line 3L, third-line. ^*∗*^Regimens received by fewer than 5 patients in the study population were grouped into this category. In the 1L setting, 33 unique regimens each were received by fewer than 5 patients. In the 2L setting, 28 unique regimens each were received by fewer than 5 patients. In the 3L setting, 27 unique regimens each were received by fewer than 5 patients. ^†^Treatment sequences received by fewer than 5 patients in the study population were grouped into this category. In total, 112 unique 1L to 2L treatment sequences were each received by fewer than 5 patients.

**Table 3:** HCRU during the study period (chart review only).

Variable	Overall (*n* = 376)	1L pre-October 2016 cohort (*n* = 97)	1L post-October 2016 cohort (*n* = 279)	*P* value	Overall (*n* = 196)	2L pre-October 2016 cohort (*n* = 77)	2L post-October 2016 cohort (*n* = 119)	*P* value
Patients hospitalized during the study period^*∗*^, *n *(%)	116 (30.9%)	57 (58.8%)	59 (21.1%)	<0.0001	82 (41.8%)	46 (59.7%)	36 (30.3%)	<0.0001
Patients with ED visits during the study period^*∗*^, *n* (%)	83 (22.1%)	44 (45.4%)	39 (14.0%)	<0.0001	60 (30.6%)	36 (46.8%)	24 (20.2%)	<0.0001
Patients with surgeries during the study period^*∗*^, *n *(%)	42 (11.2%)	22 (22.7%)	20 (7.2%)	<0.0001	32 (16.3%)	20 (26.0%)	12 (10.1%)	0.0033
Patients with outpatient visits during the study period, *n *(%)	297 (79.0%)	83 (85.6%)	214 (76.7%)	0.0649	169 (86.2%)	70 (90.9%)	99 (83.2%)	0.1258
Patients with laboratory procedures during the study period, *n *(%)	266 (70.7%)	75 (77.3%)	191 (68.5%)	0.0985	154 (78.6%)	65 (84.4%)	89 (74.8%)	0.1087
Patients who received growth factors, *n* (%)								
G-CSF	143 (38.0%)	70 (72.2%)	73 (26.2%)	<0.0001	102 (52.0%)	57 (74.0%)	45 (37.8%)	<0.0001
ESA	18 (4.8%)	8 (8.2%)	10 (3.6%)	0.0639	16 (8.2%)	8 (10.4%)	8 (6.7%)	0.3598
Patients who received dexrazoxane, *n *(%)	13 (3.5%)	2 (2.1%)	11 (3.9%)	0.5280	9 (4.6%)	2 (2.6%)	7 (5.9%)	0.4869

^*∗*^Variable assessed among patients selected for chart review (*n* = 211). 1L, first-line; 2L, second-line; ED, emergency department; ESA, erythropoietin-stimulating agents; G-CSF, granulocyte-colony stimulating factor; Min, minimum; Max, maximum; SD, standard deviation.

## Data Availability

The health data used to support the findings of this study are restricted by the US Oncology Institutional Review Board in order to protect patient privacy. For this reason, data used to support the findings of this study have not been made available.

## References

[1] NCCN (2019). *Clinical Practice Guidelines in Oncology: Soft Tissue Sarcoma*.

[2] Siegel R. L., Miller K. D., Jemal A. (2018). Cancer statistics, 2018. *CA: A Cancer Journal for Clinicians*.

[3] Sheng J. Y., Movva S. (2016). Systemic therapy for advanced soft tissue sarcoma. *Surgical Clinics of North America*.

[4] Sleijfer S., Seynaeve C., Verweij J. (2005). Using single-agent therapy in adult patients with advanced soft tissue sarcoma can still be considered standard care. *The Oncologist*.

[5] National Cancer Institute (2018). *Adult Soft Tissue Sarcoma Treatment (PDQ®): Health Professional Version, PDQ Cancer Information Summaries*.

[6] Katz D., Palmerini E., Pollack S. M. (2018). More than 50 subtypes of soft tissue sarcoma: paving the path for histology-driven treatments. *American Society of Clinical Oncology Educational Book*.

[7] American Cancer Society: Key Statistics for Soft Tissue Sarcomas

[8] ln G. K., Hu J. S., Tseng W. W. (2017). Treatment of advanced, metastatic soft tissue sarcoma: latest evidence and clinical considerations. *Therapeutic Advances in Medical Oncology*.

[9] Drugs Approved for Soft Tissue Sarcoma

[10] Wagner M. J., Amodu L. I., Duh M. S. (2015). A retrospective chart review of drug treatment patterns and clinical outcomes among patients with metastatic or recurrent soft tissue sarcoma refractory to one or more prior chemotherapy treatments. *BMC Cancer*.

[11] Villalobos V. M., Byfield S. D., Ghate S. R. (2017). A retrospective cohort study of treatment patterns among patients with metastatic soft tissue sarcoma in the US. *Clinical Sarcoma Research*.

[12] LARTRUVO, Olaratumab Prescribing Information, 2018

[13] Tap W. D., Jones R. L., Van Tine B. A. (2016). Olaratumab and doxorubicin versus doxorubicin alone for treatment of soft-tissue sarcoma: an open-label phase 1b and randomised phase 2 trial. *The Lancet*.

[14] Tap W. D., Wagner M. J., Papai Z. (2019). Announce: a randomized, placebo (PBO)-controlled, double-blind, phase (Ph) III trial of doxorubicin (dox) + olaratumab versus dox + PBO in patients (pts) with advanced soft tissue sarcomas (STS). *Journal of Clinical Oncology*.

[15] The US Oncology Network, 2018

[16] Chen C., Borker R., Ewing J. (2014). Epidemiology, treatment patterns, and outcomes of metastatic soft tissue sarcoma in a community-based oncology network. *Sarcoma*.

[17] Ray-Coquard I., Collard O., Ducimetiere F. (2017). Treatment patterns and survival in an exhaustive French cohort of pazopanib-eligible patients with metastatic soft tissue sarcoma (STS). *BMC Cancer*.

[18] Nagar S. P., Mytelka D. S., Candrilli S. D. (2018). Treatment patterns and survival among adult patients with advanced soft tissue sarcoma: a retrospective medical record review in the United Kingdom, Spain, Germany, and France. *Sarcoma*.

[19] Meyer M., Seetharam M. (2019). First-line therapy for metastatic soft tissue sarcoma. *Current Treatment Options in Oncology*.

[20] Bleloch J. S., Ballim R. D., Kimani S. (2017). Managing sarcoma: where have we come from and where are we going?. *Therapeutic Advances in Medical Oncology*.

[21] Seddon B., Strauss S. J., Whelan J. (2017). Gemcitabine and docetaxel versus doxorubicin as first-line treatment in previously untreated advanced unresectable or metastatic soft-tissue sarcomas (GeDDiS): a randomised controlled phase 3 trial. *The Lancet Oncology*.

[22] Tap W. D. (2017). GeDDiS: insight into frontline therapy in soft tissue sarcoma. *The Lancet Oncology*.

